# Only peak thyroglobulin concentration on day 1 and 3 of rhTSH-aided RAI adjuvant treatment has prognostic implications in differentiated thyroid cancer

**DOI:** 10.1007/s12149-021-01663-y

**Published:** 2021-08-07

**Authors:** Aleksandra Ledwon, Ewa Paliczka-Cieślik, Aleksandra Syguła, Tomasz Olczyk, Aleksandra Kropińska, Agnieszka Kotecka-Blicharz, Kornelia Hasse-Lazar, Aneta Kluczewska-Gałka, Barbara Jarząb, Daria Handkiewicz-Junak

**Affiliations:** Department of Nuclear Medicine and Endocrine Oncology, Maria Sklodowska-Curie National Research Institute of Oncology, Gliwice Branch, Gliwice, Poland

**Keywords:** Differentiated thyroid carcinoma (DTC), Thyroglobulin (Tg), Radioiodine (RAI), Recombinant human thyroid-stimulating hormone (rhTSH), Prognostic marker, Structural recurrence

## Abstract

**Objective:**

In patients with differentiated thyroid carcinoma (DTC), serum thyroglobulin levels measured at the time of remnant ablation after thyroid hormone withdrawal were shown to have prognostic value for disease-free status. We sought to evaluate serial thyroglobulin measurements at the time of recombinant human thyroid-stimulating hormone (rhTSH)-aided iodine 131 (^131^I) adjuvant treatment as prognostic markers of DTC.

**Methods:**

Six hundred-fifty patients with DTC given total/near-total thyroidectomy and adjuvant radioiodine post-rhTSH stimulation were evaluated. Thyroglobulin was measured on day 1 (Tg1; at the time of the first rhTSH injection), day 3 (Tg3; 1 day after the second, final rhTSH injection), and day 6 (Tg6; 3 days post-radioiodine administration). Treatment failure was defined as histopathologically confirmed locoregional recurrence, or radiologically-evident distant metastases (signs of disease on computer tomography (CT) or magnetic resonance imaging (MRI), or abnormal foci of radioiodine or [^18^F] fluorodeoxyglucose ([^18^F]FDG) uptake.

**Results:**

In univariate analysis, Tg1 (*p* < 0.001) and Tg3 (*p* < 0.001), but not Tg6, were significantly associated with structural recurrence. In multivariate analysis of the overall cohort, only Tg3 was independently associated with structural recurrence. In multivariate analysis of the subgroup (*n* = 561) with anti-Tg antibodies titers below the institutional cut-off, 115 IU/mL, Tg1 was an independent prognostic marker. Tg1 and Tg3 cutoffs to best predict structural recurrence were established at 0.7 ng/mL and 1.4 ng/mL, respectively.

**Conclusions:**

Tg1 and Tg3, measurements made after rhTSH stimulation but before radioiodine treatment, independently predict a low risk of treatment failure in patients with DTC. Levels measured post-radioiodine application (e.g., Tg6) are highly variable, lack prognostic value, and hence can be omitted.

**Supplementary Information:**

The online version contains supplementary material available at 10.1007/s12149-021-01663-y.

## Introduction

In patients with differentiated thyroid cancer (DTC), stimulated serum thyroglobulin (Tg) concentration measured around the time of radioiodine (RAI) treatment is a valuable prognostic factor for persistent/recurrent disease [1–5]. Given the analyte’s approximately 65-h half-life, serum Tg levels should in theory drop to undetectable approximately 1 month after total thyroidectomy. However, even after such surgery, Tg level is variable and may depend on thyroid remnant volume and thyroid-stimulating hormone (TSH) concentration. Tg cutoff values are also dependent on the TSH stimulation method. Stimulated Tg cutoffs have mainly been established in patients undergoing thyroid hormone withdrawal (THW) [6–8]. In contrast, data on optimal measurement times and on the prognostic value of the Tg level after recombinant human TSH (rhTSH) stimulation are rather limited [9–12]. Only one report has compared measurements taken at different points around the time of rhTSH stimulation [13]. In diagnostic settings, the highest prognostic value of Tg measurement is 5 days after the first rhTSH injection [14, 15]. However, in the adjuvant RAI therapy setting, concern has been raised because Tg levels are affected not only by residual cancer cells but mostly by remnant thyrocytes, especially after RAI-induced thyrocyte damage [16]. On the other hand, some studies showed that the ratio of pre-RAI (at RAI administration) to post-RAI (5 days post-therapy) Tg measurements is of prognostic significance [17]. The question of when is the best time to measure Tg during rhTSH-aided adjuvant RAI treatment remains open.

After introducing rhTSH-aided adjuvant RAI treatment in patients with DTC, we selected Tg measurement on day 1, 3, and 6 starting with the first rhTSH injection. However, as our clinical experience with rhTSH grew, we noticed that in patients with thyroid remnant, in contrast to those receiving repeated treatment for metastases, Tg6 appeared to be highly variable and not to correlate with clinical outcome*. *The present study was undertaken to evaluate in a large cohort of patients with DTC the optimal time(s) during rhTSH-aided adjuvant RAI therapy to measure Tg concentration. We also sought to define the prognostic significance of Tg measurements during this period.

## Materials and methods

We retrospectively reviewed records of patients who underwent adjuvant RAI treatment after rhTSH stimulation from 2008 to 2011. Six hundred-fifty patients with DTC after total/near-total thyroidectomy, without persistent disease and treated for the first time with rhTSH-aided RAI, were included in the analysis. Cases of persistent disease were excluded based on neck ultrasound, post-therapy scintigraphy, and/or chest x-ray. In case of elevated (> 10 ng/mL) Tg during TSH suppression, neck and chest CT was done, and if this imaging was negative, patients were also included in the study. During radioiodine therapy, thyroid remnant volume on neck ultrasound performed on day 1 of rh-TSH stumulation was < 2 mL in all except five patients. In those individuals, due to additional medical conditions, RAI therapy was given without completion thyroidectomy. Patient characteristics are summarized in Table 1.

**Table 1:** Patient characteristics

Characteristic		Whole group of patients (*N* = 650) (%)	Subgroup with TgAbs below institutional cutoff (*n* = 561) (%)
Gender: female/male		535 (82.3)/115 (17.7)	455 (81.1)/106 (18.9)
Age (yr): median (minimum–maximum)		53 (13–85)	54 (16–86)
Total/near-total thyroidectomy		595 (91.5)/ 55 (8.5)	511 (92)/50 (8)
Central lymph node dissection		341 (52.5)	288 (51)
Lateral lymph node dissection		68 (10.5)	56 (10)
TNM classification (7th edition)			
Primary tumor			
T0		3 (0.5)	3 (0.5)
T1		362 (55.7)	310 (55.3)
T2		82 (12.6)	72 (12.8)
3		142 (21.8)	123 (21.9)
Intrathyroidal tumor > 4 cm in greatest dimension		26 (4)	25 (4.4)
Extrathyroidal extension		116 (17.8)	98 (17.5)
T4		7 (1.1)	4 (0.8)
Tx		54 (8.3)	49 (8.7)
Lymph node			
N0		241 (37.1)	208 (37.1)
N1		137 (21.1)	109 (19.4)
N1a		74 (11.4)	59 (10.5)
N1b		63 (9.7)	50 (8.9)
Nx		272 (41.8)	244 (43.5)
Histology			
Papillary		577 (88.8)	495 (88.2)
Papillary–aggressive variant^a^		15 (2.3)	12 (2.1)
Follicular		52 (8)	48 (8.6)
Poorly differentiated		6 (0.9)	6 (1.1)
Multifocality		153 (23.5)	129 (23)

TgAbs anti-thyroglobulin antibodies

^a^Includes solid variant, tall cell variant, columnar cell variant, and diffuse sclerosing variant

rhTSH application as preparation for RAI therapy was approved by the Ethics Review Committee of our institution. As a retrospective analysis in which all patient information was de-identified, this study was determined to be exempt from approval by our Institutional Review Board.

## Treatment protocol and follow-up

According to institutional guidelines at the time of treatment, all patients, except those with pT1aN0M0 papillary thyroid cancer, were referred for RAI adjuvant therapy. Median time from surgery to such therapy was 80 (13–370) days, and median administered RAI activity was 3.7 GBq [100 mCi] (minimum–maximum: 3.7–5.55 GBq [100–150 mCi]). All patients were hospitalized in a radionuclide therapy department with full radiation protection for at least 3 days after RAI administration, then discharged when the radiation dose rate at 1 m was < 20 mSv/h.

Tg measurement was performed on day 1 (Tg1; just before the first injection of rhTSH, 0.9 mg), on day 3 (Tg3; one day after the second, final injection of rhTSH, 0.9 mg), and on day 6 (Tg6; 48 and 120 h, respectively, after the first rhTSH injection, and three days after RAI administration). Seventy-two hours after ^131^I administration, whole-body scintigraphy (WBS) was performed (Supplementary Fig. 1). When RAI uptake outside the thyroid bed was suspected, single-photon emission computed tomography fusion imaging (SPECT/CT) was used to clarify the region of uptake.

After RAI treatment, all patients were followed-up with neck ultrasound and TSH, Tg, and anti-Tg antibodies (TgAbs) determinations at 6-month to 18-month intervals. Other examinations were performed as indicated. TSH level was kept within 0.1–0.4 uIU/mL. Stimulated Tg and diagnostic ^131^I WBS was performed 12–24 months after RAI treatment to assess that modality’s efficacy. Median follow-up for the study sample was 6 years (minimum–maximum: 5–8 years).

## Biochemical measurements

Serum Tg was measured via chemiluminescence assay (Roche Diagnostic, Meylan, France), which during the time of treatment, had an analytical sensitivity of 0.1 ng/mL l or 0.04 ng/ml (from 2014) and functional sensitivity < 1 ng/mL. Serum TgAbs were measured with the Roche Diagnostic Elecsys assay, with an analytical sensitivity of 10 IU/mL and a reference value of 10–4000 IU/mL.

## Imaging methods

Ultrasound was performed with a linear multifrequency 10-MHz transducer for morphological analysis and for Doppler evaluation. All suspicious findings were submitted to ultrasound-guided fine-needle aspiration biopsy.

Post-therapy WBS and, when required, spot or SPECT imaging, were performed 72 h after ^131^I treatment, with a dual-head gamma camera (Multispect 2 or E.Cam-Duet, Siemens, Erlangen, Germany) equipped with parallel high-energy collimators. All non-physiological areas of iodine uptake outside the thyroid bed were considered to be positive findings for DTC metastases, and patients were not included in the study.

## Definition and diagnosis of treatment failure

For the purpose of the study, only structural recurrences were considered as treatment failure. If structural recurrence was suspected on neck ultrasound, fine-needle biopsy was performed, with histopathological evidence of such recurrence considered to be confirmatory. At sites outside the neck, CT, MRI or [^18^F]FDG PET/CT were the gold standard to confirm metastatic disease. The indication for radiological examination was an increasing Tg level or abnormal findings in clinical examinations.

## Statistics

Quantitative data are expressed as mean ± SD (standard deviation). Between-group differences were assessed by two-tailed unpaired *t* test. The predictive value of Tg with respect to different clinical variables was assessed by univariate and multivariate Cox proportional hazards modeling. The cutoff values to optimally predict structural recurrence for Tg measured at different time intervals during RAI treatment were selected by analyzing Receiver Operating Characteristic (ROC) curves. Diagnostic performance (sensitivity, specificity, positive predictive value [PPV] and negative predictive value [NPV]) of Tg was evaluated based on the cutoff values obtained by ROC curve analysis. Kaplan-Mayer curves were used for survival analysis. R software (R Foundation, Vienna, Austria) was used for statistical analysis. Statistical significance was defined by a *p* value < 0.05.

## Results

### Biochemical evaluation

At the time of RAI treatment, TgAbs were detectable in 343 patients (53%) and were above the institutional cutoff of 115 IU/mL in 89 (14%). There was a statistically significant negative correlation between Tg concentration and TgAbs level, but the coefficient of correlation was very weak: − 0.179 on day 1, − 0.180 on day 3, and − 0.135 on day 6 (Supplementary Fig. 2). Due to this weak but significant correlation, we performed all analyses first in the whole group of patients (*N* = 650) and then in a subgroup with TgAbs below the institutional cutoff (*n* = 561).

Median Tg concentration on day 1, day 3, and day 6 increased from 0.2 ng/mL to 1.0 ng/mL to 6.0 ng/mL, respectively (Table 2). The median stimulated Tg value was highest on day 6 of stimulation (72 h after RAI application).

**Table 2:** Tg concentration on day 1, day 3, and 6

Day of stimulation^a^	Number of Tg evaluations above detection threshold^a^ (%)	Number of determinations of Tg level > 10 ng/mL (%)	Median (minimum–maximum) Tg level (ng/mL)	Median)(minimum–maximum)Tg level for Tg evaluation above institutional detection threshold^†^ (ng/mL)
Day 1	393 (60.5)	8 (1.2)	0.2 (0.1–75.3)	0.53 (0.1–75.3)
Day 3	508 (78)	80 (12)	1 (0.1–03)	1.5 (0.1–303)
Day 6	592 (91)	278 (43)	6 (0.1–1492)	7.7 (0.1–1492)

*RAI* radioiodine, *rhTSH* recombinant human thyroid-stimulating hormone, *Tg* thyroglobulin

^a^The day 1 sample was taken immediately before the first injection of rhTSH, and the day 3 and day 6 samples were taken 24 and 96 h, respectively, after the second and final injection of rhTSH, and just before and72 hours, respectively, after RAI administration

^**†**^The institutional detection threshold for Tg was 0.1 ng/mL

There was a highly significant correlation between Tg level and thyroid remnant volume on ultrasound performed on the first day of rh-TSH stimulation (Table 3). In patients with thyroid remnant volume > 1 mL, the median Tg concentration increased more than 63.6-fold on day 6.

**Table 3:** Tg concentration in relation to thyroid remnant volume

Thyroid remnant volume^a^	Day of rhTSH stimulation	Tg, mean ± SD		Tg, median (minimum–maximum)	*p* value
None (*n* = 521)	1	0.6	± 1.6	0.2 (< 0.1–17.9)	*p* < 0.0001
	3	2.8	± 7.3	0.6 (< 0.1–77.8)	
	6	25.8	± 61.8	4.6 (< 0.1–494)	
≤ 1 mL (*n* = 94)	1	1.4	± 2.9	0.5 (< 0.1–23.9)	*p* < 0.0001
	3	5.8	1 ± 1.2	2.0 (< 0.1–99.3)	
	6	77.9	± 137.0	25.0 (< 0.1–682.6)	
> 1 ml (*n* = 35)	1	6.4	± 15.8	1.5 (< 0.1–75)	*p* < 0.0001
	3	29.4	± 57.2	14.4 (< 0.1–302)	
	6	260.8	± 333.4	95.4 (< 0.1–1492)	

*rhTSH* recombinant human TSH, *Tg* thyroglobulin

^a^Volume was determined by neck ultrasound performed on day 1 of rh-TSH stimulation

### Risk of thyroid cancer structural recurrence

**Fig. 1:**
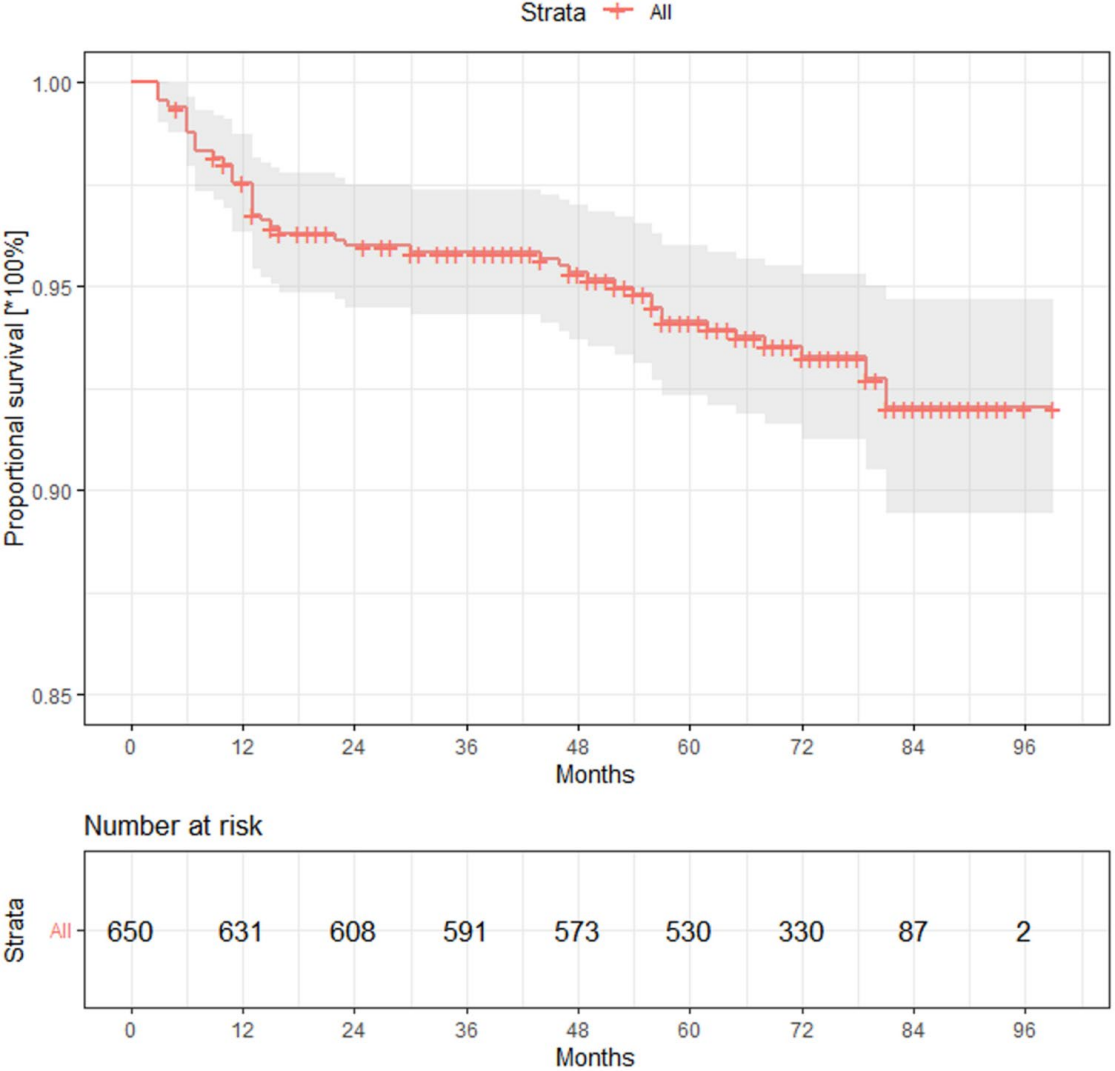
Progression (structural recurrence)-free survival (PFS) in the whole group of patients (*N* = 650). Dotted lines represent the 95% confidence interval (95% CI)

After a respective median follow-up of 6 years, structural recurrence was observed in 43/650 patients (6.6%) (Fig. 1) in the overall study sample and in 38/561 patients (6.8%) with TgAbs below the institutional cutoff. Median time to structural recurrence was 13 months. The recurrence rate was 6%, 9%, and 18%, respectively in the whole group of patients after 2, 3, and 5 years of follow-up. Most relapses (34/43, 79%) were found in the neck, 8 (19%) were distant metastases, and one patient was diagnosed with both local recurrence and distant metastases.

**Fig. 2:**
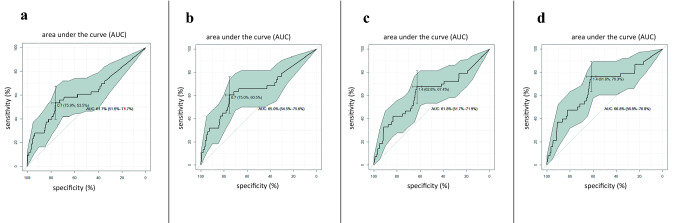
Receiver–operating characteristic curve analysis for Tg1 (Tg measured on day 1) in the whole group of patients (**a**) and in patients with anti-Tg antibodies (TgAbs) below the institutional cutoff (**b**), and for Tg3 in the whole group of patients (**c**) and in patients with TgAbs below the institutional cutoff (**d**)

In patients with TgAbs below the institutional cutoff, the recurrence rate was 10%, 13% and 22%, respectively, after 2, 3, and 5 years of follow-up.

In another 6/650 patients (0.9%), stable elevated Tg (> 1 ng/mL) was detected, but since these individuals were not diagnosed with structural recurrence, these cases were not considered treatment failures.

### Univariate and multivariate analysis for the risk of structural recurrence

#### The whole group of 650 patients (patients with detectable TgAbs included)

In the univariate analyses, only Tg levels measured on day 1 (Tg1) (*p* < 0.001) and day 3 (Tg3) (*p* < 0.001) were significantly associated with structural recurrence. Such correlation was not observed for Tg level measured on day 6. Among the analyzed biochemical factors, TSH concentration measured 24 h after the second injection of rhTSH (TSH3) was also significant for the risk of structural recurrence. In addition, age at diagnosis, sex, histology, presence of extrathyroidal extension, tumor size, presence of lateral lymph node metastases, and history of lateral lymph node dissection were found to be statistically significantly associated with structural recurrence in univariate analyses. The detailed results of these analyses are summarized in Table 4.

**Table 4:** Univariate analysis of entire cohort (*N* = 650): significant prognostic factors for structural recurrence

Prognostic factor	Hazard ratio	95% confidence interval	*p* value
Papillary	1.00	(Ref.)	
Follicular	2.44	(1.08–5.53)	0.032
Poorly differentiated	6.42	(1.54–26.8)	0.011
pT1	1.00	(Ref.)	
pT2	3.93	(1.43–10.9)	0.0081
pT3/pT4 (extrathyroidal extension)	6.96	(3.03–16.0)	< 0.0001
pTx	6.28	(2.27–17.3)	0.0004
pN0	1.00	(Ref.)	
pN1b	4.84	(2.09–11.2)	0.0002
Female	0.42	(0.22–0.80)	0.0081
Age (per additional yr)	1.03	(1.01–1.06)	0.0048
Lateral lymph node dissection	4.02	(2.1–7.70)	< 0.0001
Tg1 (ng/ml)	1.06	(1.03–1.08)	< 0.0001
Tg3 (ng/ml)	1.02	(1.01–1.023)	< 0.0001
TSH3 (mIU/ml)	1.01	1.002–1.011)	0.0094

Tg6 was insignificant prognostic factor. ALL TNM classifications were according to the 7th edition

*Ref.* reference, *Tg* thyroglobulin, *Tg1* thyroglobulin measured on day 1, *Tg3* thyroglobulin measured on day 3, *Tg6* thyroglobulin measured on day 6, *TSH3* TSH measured on day 3

In the multivariate analysis, studied factors independently associated with structural recurrence were: Tg level measured on day 3, age at diagnosis, male gender, and history of lateral lymph node dissection. Tg concentration on day 6 was not significant (Table 5).

**Table 5:** Multivariate analysis of entire cohort (*N* = 650): significant prognostic factors for structural recurrence

Prognostic factor	Hazard ratio	95% confidence interval	*p* value
Female	0.51	(0.26–0.97)	0.0414
Age (per additional yr)	1.04	(1.02–1.06)	0.0003
Lateral lymph node dissection	5.32	(2.67–10.61)	< 0.0001
Tg3 (ng/ml)	1.016	(1.009–1.024)	< 0.0001

Tg1 was insignificant prognostic factor

*Tg1* thyroglobulin measured on day 1, *Tg3* thyroglobulin measured on day 3

#### Patients with negative TgAbs

Similarly to the whole group of patients, in the subgroup negative for TgAbs, only Tg levels measured on day 1 and day 3 were significantly associated with structural recurrence. In multivariate analysis, Tg1 was the only independent prognostic marker (Table 6).

**Table 6:** Multivariate analysis of subgroup with TgAbs below institutional cutoff level (*n* = 561): significant prognostic factors for structural recurrence

Prognostic factor	Hazard ratio	95% confidence interval	*p* value
Papillary	1.00	(Ref.)	
Poorly differentiated	11.89	(2.61–54.2)	0.0014
pT1	1.00	(Ref.)	
pT2	3.11	(1.03–9.37)	0.0435
pT3/pT4 (extrathyroidal extension)	4.83	(2.01–11.6)	0.0004
Age (per additional yr)	1.04	(1.02–1.07)	0.0016
Lateral lymph node dissection	5.27	(2.46–11.83)	< 0.0001
Tg1 (ng/ml)	1.05	(1.02–1.08)	< 0.0021

Tg3 was insignificant prognostic factor. ALL TNM classifications were made according to the 7th edition

*Tg1* thyroglobulin measured on day 1, *Tg3* thyroglobulin measured on day 3

### Analysis of ROC curves for optimal Tg concentration cutoff

In the whole group of patients, in ROC curve analysis of the optimal cutoff to predict structural recurrence of DTC, the cutoff for Tg1 was 0.7 ng/mL. The area under the curve (AUC), a measure of the cutoff’s prognostic performance, was 61.7% (95% CI, 51.6%–71.7%). Using this cutoff, Tg1 had a sensitivity of 53.5%, a specificity of 75.9%, an NPV of 96%, and a PPV of 14% for predicting structural recurrence.

The cutoff of Tg3 was 1.4 ng/mL and the AUC was 61.8%(95% CI, 51.7%–71.9%). using this cutoff, Tg3 had a sensitivity of 67.4%, a specificity of 62.8%, an NPV of 96%, and a PPV of 11% for predicting structural recurrence.

Results for the subgroup with TgAbs below 115 IU/mL (*n* = 591) did not differ from those in the whole group of patients. The ROC curves are shown in Fig. 2. 

## Discussion

In the present study, we showed that during rhTSH-aided adjuvant RAI treatment of DTC, Tg concentration after RAI application is highly variable and does not have prognostic significance. However, Tg concentration measured immediately before the first rhTSH injection or 24 h after the second rhTSH injection are independent prognostic factors, which allow selection of the group of patients with very good prognosis and low risk of structural recurrence. We focused only on structurally recurrent disease to choose the group of patients with unequivocal treatment failure that necessitates therapeutic intervention. Since most structural recurrences occur during the first 5 years after DTC diagnosis, the follow-up time in our study was long enough to pick up most such cases.

In the interpretation of postoperative Tg concentration, the time from surgery to ^131^I therapy is very important. The three main reasons for this importance are: (1) the half-life of Tg released into the blood during surgery, (2) the ability of the thyroid remnants to produce and release Tg, and (3) the resolution of postoperative edema, which impedes the assessment of thyroid remnants and persistent neck disease. In our study sample, the median time from surgery to RAI therapy was 68 days, so such treatment took place at an optimal time for wound healing, Tg clearance from the blood stream, and detection of persistent disease.

Several studies in patients prepared with THW have demonstrated that stimulated Tg level measured just before RAI has prognostic significance [4, 17–19] and currently, the measurement of serum Tg at the time of adjuvant RAI treatment is suggested in most DTC guidelines [3, 20]. In the diagnostic setting, Tg measurement 72 h after the second rhTSH injection is usually recommended, because that timepoint corresponds to the Tg peak level [21] and is predictive for persistent/recurrent disease. It would seem reasonable to have the same approach during RAI treatment, however, Tg released due to thyrocyte RAI damage may confound interpretation of the results [16].

Some studies demonstrated that Tg measurement after rhTSH stimulation and therapeutic RAI application is of prognostic significance [9, 11, 13, 22]. Melo et al. [11] reported the prognostic value of Tg5 (i.e., measurement 5 days after first rhTSH injection) in predicting disease status 1 year post-ablation. The cutoff value of Tg5, 7.2 ng/mL, was associated with an NPV of 89.6%, and was demonstrated to be an independent prognostic marker. In 2016 Moon et al. [9] published results confirming the utility of Tg5 measurement at ablation in patients after total thyroidectomy and prophylactic central neck dissection. The optimal cutoff value was 1.79 ng/mL, with an NPV of 99.5%. The difference between the above-mentioned cutoff values of Tg5 was explained by the presence or absence of prophylactic neck dissection and by surgeon experience in total thyroidectomy. Also recently, Mutstudy et al. [13] showed that Tg levels measured two days after RAI therapy have prognostic significance. ROC curve analysis showed an optimal Tg cutoff value of 3.7 ng/mL, which was confirmed as an independent prognostic factor in multivariate analysis. However, in contrast to our study, follow-up time in those studies was rather short (9–18 months) and usually both structural and biochemical failures with Tg > 1 ng/mL were included.

Our results agree with work showing that RAI therapy-induced thyrocyte damage and released Tg into the bloodstream. Some authors assume [5] that the devascularized normal thyroid remnant is less capable of producing and releasing Tg in comparison to lymph node metastases. In our study, Tg6 showed a very strong correlation with thyroid remnants volume in neck ultrasound performed on first day of rh-TSH stimulation, and was also high in patients with thyroid remnants diagnosed in neck RAI scan but not in ultrasound [23]. In the “real world”, thyroid cancer patients are not always operated on in hospitals with expertise in thyroid surgery, and to decrease the risk of surgical complications, some thyroid remnants are left in place. In our study, Tg concentration was the highest 72 h after ^131^I treatment. However, in univariate analysis, the Tg concentration at that time was non-significant as a prognostic factor for structural recurrence. Of interest is the fact, that in multivariate analysis of the whole group of patients, Tg3 concentration was a significant prognosticator of recurrence, but in patients with TgAbs below the institutional cutoff, Tg1 was statistically significant. One cannot exclude, that in the whole group of patients, Tg1 could be underestimated as a result of TgAbs interference in Tg measurement by immunometric assays. Higher Tg3 values after TSH stimulation can reduce the effect of antibody positivity since the increasing Tg concentration on day 3 (24 h after the second injection of rhTSH) could diminish the degree of the interference in Tg measurement. The propensity for TgAbs interference is known to be lowest when Tg levels are higher [24].

Tg cutoffs on day 1 and day 3 of rhTSh stimulation were 0.7 ng/mL and 1.4 ng/mL, respectively, which correspond to results from other studies [10, 12]. However, it should be underlined, that Tg concentration below this cutoff value has a very high NPV (about 96%), but PPV for higher Tg concentrations is rather low. In a majority of patients, elevated Tg levels decline over time [25], which could explain the low PPV of Tg measurement during preparation for RAI therapy. Higer cutoff values would result in higher PPV, however, with much lower sensitivity in this group of patients with a relatively good prognosis. From a clinical practice point of view, it means that in patients with low Tg concentration on day 1 and day 3 of rhTSH stimulation (before RAI application), we can have less intensive surveillance.

This study has some limitations related to its retrospective data collection. Nevertheless, it also has three important strengths. First, data were collected from consecutive patients treated during three years. Second, only patients without persistent disease in ultrasonographic, radiological, and scintigraphic evaluation were enrolled. Third, the high number of patients allowed the performance of reliable statistical analysis.

In conclusion, serum Tg levels measured on day 1 and day 3 of rhTSH stimulation (before RAI treatment) independently predict a low risk of structural recurrence of DTC. Tg measured shortly after RAI application is highly variable, has no prognostic value, and hence can be avoided.

## Supplementary Information

Below is the link to the electronic supplementary material.

Supplementary Fig. 1. rhTSH aided adjuvant radioiodine protocol. WBS – whole body scan. Tg – thyroglobulin. TgAbs – anti-Tg antibodies. TSH – thyroid stimulating hormone (PDF 169 KB)

Supplementary Fig. 2. Correlation between thyroglobulin concentration (Tg) and anti-thyroglobulin antibodies (TgAbs) level (PDF 43 KB)
