# Supplementary figures and images for: Only peak thyroglobulin concentration on day 1 and 3 of rhTSH-aided RAI adjuvant treatment has prognostic implications in differentiated thyroid cancer
Source: Ann Nucl Med. 2021 Aug 7;35(11):1214–22. doi: 10.1007/s12149-021-01663-y (PMC8494717; doi:10.1007/s12149-021-01663-y)

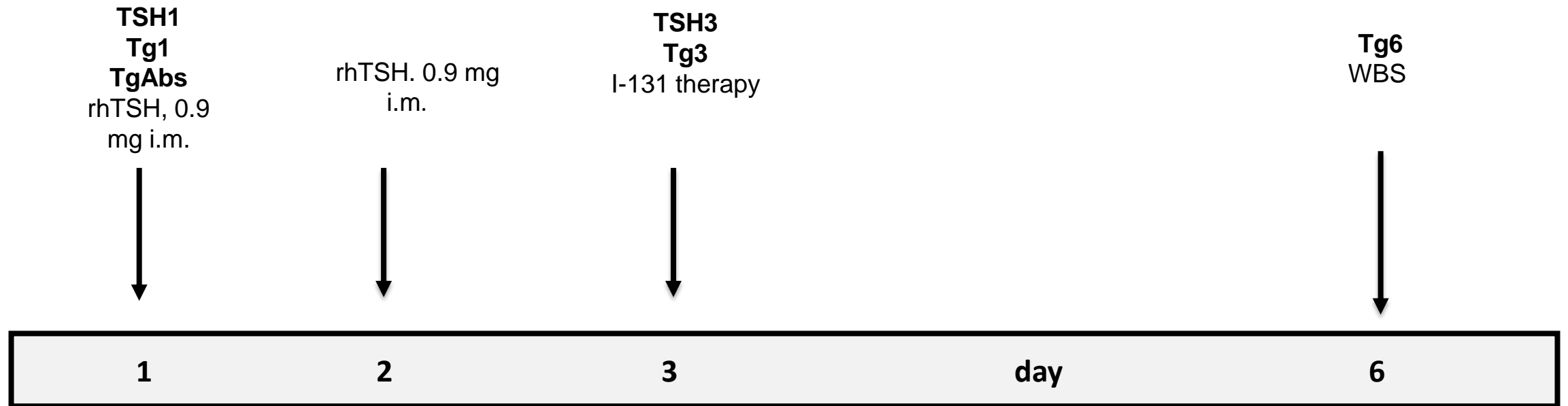

Supplement: Supplementary file 1 — Supplementary Fig. 1. rhTSH aided adjuvant radioiodine protocol. WBS – whole body scan. Tg – thyroglobulin. TgAbs – anti-Tg antibodies. TSH – thyroid stimulating hormone (PDF 169 KB) [file 12149_2021_1663_MOESM1_ESM.pdf]

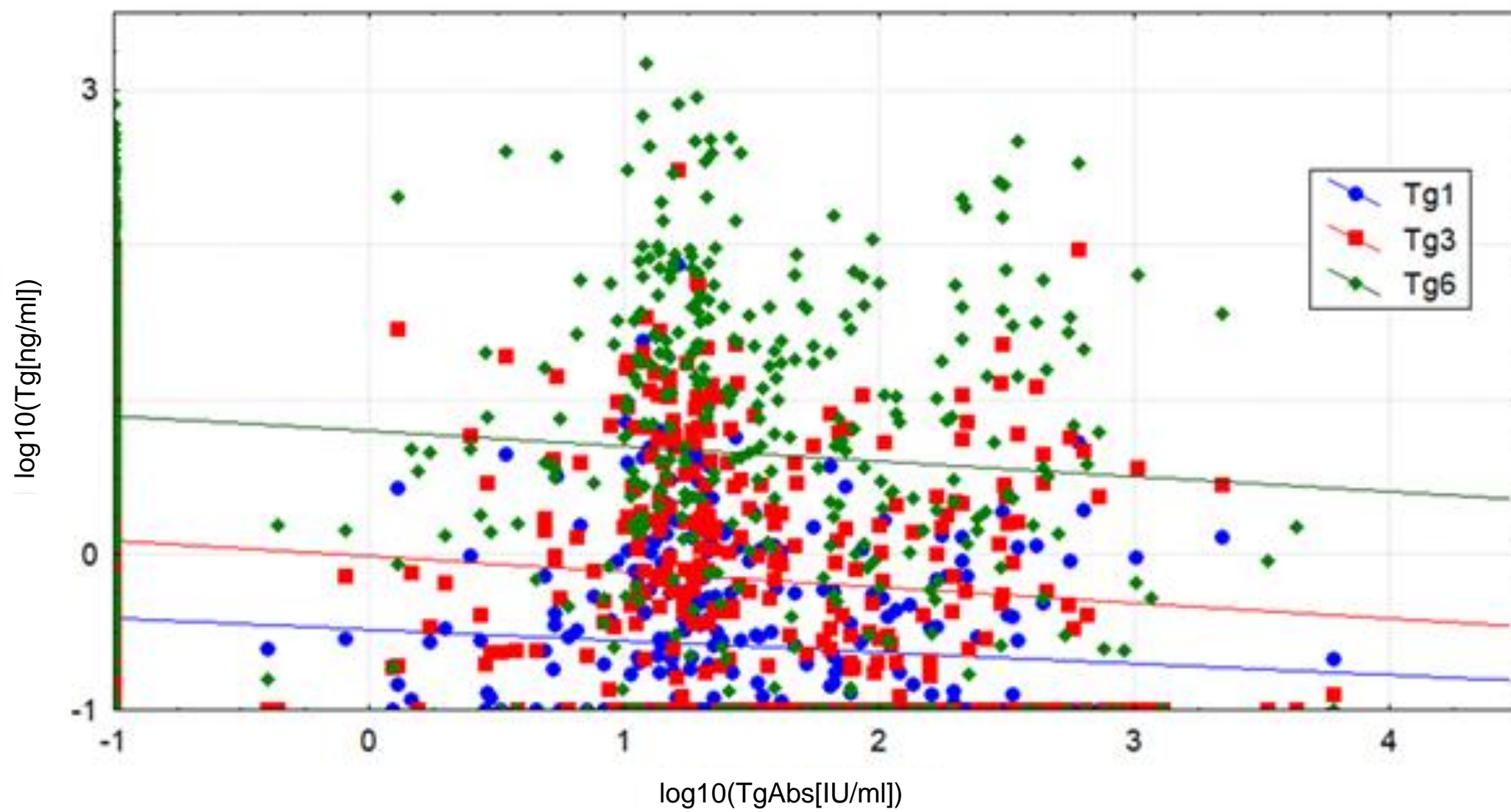

Supplement: Supplementary file 2 — Supplementary Fig. 2. Correlation between thyroglobulin concentration (Tg) and anti-thyroglobulin antibodies (TgAbs) level (PDF 43 KB) [file 12149_2021_1663_MOESM2_ESM.pdf]
